# Utilizing Baidu Index to Investigate Seasonality, Spatial Distribution and Public Attention of Dry Eye Diseases in Chinese Mainland

**DOI:** 10.3389/fpubh.2022.834926

**Published:** 2022-07-06

**Authors:** Haozhe Yu, Weizhen Zeng, Mengyao Zhang, Gezheng Zhao, Wenyu Wu, Yun Feng

**Affiliations:** ^1^Department of Ophthalmology, Peking University Third Hospital, Beijing, China; ^2^Department of Ophthalmology, Peking University Third Hospital Yanqing Hospital, Beijing, China; ^3^School of Nursing, Peking University, Beijing, China

**Keywords:** Dry eye diseases, Baidu index, prevalence, infodemiology, DED

## Abstract

**Purpose:**

To explore the characteristics of spatial-temporal prevalence and public attention of dry eye diseases (DED) through Baidu Index (BI) based on infodemiology method.

**Methods:**

The data about BI of DED were collected from Baidu search engine using “Dry eye diseases” as keyword. The spatial and temporal distribution of DED were analyzed through timeseries data decomposition as well as spatial autocorrelation and hotspot detection of BI about DED. The most popular related words and demographic characteristics were recorded to determine the public attention of DED.

**Results:**

The trends of BI about DED in Chinese mainland had gradually increased over time with a rapid increase from 2012 to 2014 and in 2018. The results of timeseries decomposition indicated that there was seasonality in the distribution of BI about DED with the peak in winter, especially in northern regions. The geographic distribution demonstrated the search activities of DED was highest in the east of Chinese mainland while lowest in the west. The vast majority of people searching for DED were teenagers (20–29 years), with a predominance of females. Glaucoma, keratitis and conjunctivitis were the diseases most often confused with DED, and the artificial tears were the most common treatment for DED in Chinese mainland according to the BI about DED.

**Conclusions:**

The analysis revealed the seasonality, geographic hotspots and public concern of DED through BI in Chinese mainland, which provided new insights into the epidemiology of DED.

## Introduction

Dry eye disease (DED) is a common disease characterized by tear film instability and hyperosmolarity, inflammation, and neurosensory abnormalities. It is estimated that the worldwide global prevalence of DED ranged from 5 to 50% according to the Tear Film and Ocular Surface Society (TFOS), which causes great burden in healthcare and socioeconomy (1). In this context, many epidemiological studies have been carried out to clarify the risk factors of the DED for developing prevention strategies (2–5). Recently, the spatiotemporal differences of the incidence and prevalence of DED were proposed, which were also considered as the most cost-effective intervention for reducing the negative effects of diseases (6–9). However, these results remain controversial limited by the small sample sizes and regional restrictions, which is generally accepted that the epidemiological studies of such high prevalence diseases are costly for developing countries to carry out (10).

With the rapid development of information technology, the internet has become the main way for people to obtain information, and it was reported that nearly 70% adolescents searched medical-related issues through internet (11). On this basis, the concept of infodemiology has been developed, which was mainly based on the internet information to conduct the analysis of diseases (12). Google Trends is considered as the most useful tool of infodemiology, and several research have predicted diseases prevalence, monitored infectious diseases and identified the public concern through analyzing Google Trends (13–16).

However, Google Trends is not available in Chinese mainland, while the Baidu is viewed as the top search engine in Chinese mainland with nearly 65 % of web surfers ranking Baidu as their first choice to seek for medical-related information, which is equal to the role of Google in western countries (17). In addition, considering the screen time has been identified as an important risk factor of DED, we assume that the population of people using Baidu to search for DED is highly overlapping with the DED patients in Chinese mainland. Therefore, this study aims to analyze the DED searching behavior of users through Baidu Index (BI) to determine its public interest and spatial-temporal distribution. Such results can provide reference for developing better interventions and treatments of DED patients and further conducting epidemiological study of DED.

## Methods

The Baidu Index (BI) is provided by Baidu Inc., which is a data sharing platform based on the searching behavior of all Chinese Mainland Baidu users (https://index.baidu.com/v2/index.html). BI can be divided into PC (Personal Computer) BI as well as Mobile BI, and the basic of BI calculation is the frequency of keywords searching in Baidu over a period of time, plus with the further filtering and weighting by their IT services technicians such as the derivation and variation word. Considering the widely known of DED and the availability of data in BI, the search term was finally identified as “Dry eye diseases.” We collected the BI about DED from January 1, 2011 to December 5, 2021 covering 31 provinces, municipalities, and autonomous regions in Chinese mainland. As the time series exceeds more than 1 year, only the 7 days average BI will be available for public. Therefore, a total of 17, 701 (571 weeks and 31 regions) sets of data were collected in this study.

Seasonality and trend analysis of BI were carried out using R 4.1.1 software based on the Holt-Winters seasonal methods. Before analysis, the time series data were smoothed by calculating their simple moving average with the k value of 15. All data were decomposed into trend factors, seasonal factors and random factors, which represented long-term changes, periodic changes and irregular changes in the data, respectively. The seasonality <0.4 was considered as no seasonal trend. The data with provinces and search trends was imported into ArcGis 10.7 software to map the spatial distribution of the BI index about DED. The Getis-Ord Gi^*^ statistic and standard deviation ellipse as well as global (Global Moran's I) and local spatial autocorrelation were used to determine the geographical trends and spatial aggregation of BI about DED. *P* < 0.05 was considered statistically significant. In addition, we collected the user demand graphs, related words popularity, and searching population portraits from the database over the period, and calculated their average by season and sorted to get the Top 10 search keywords for further understanding the public interest in DED.

## Results

### Searches Trends and Seasonal Variation of DED

**Figure 1:**
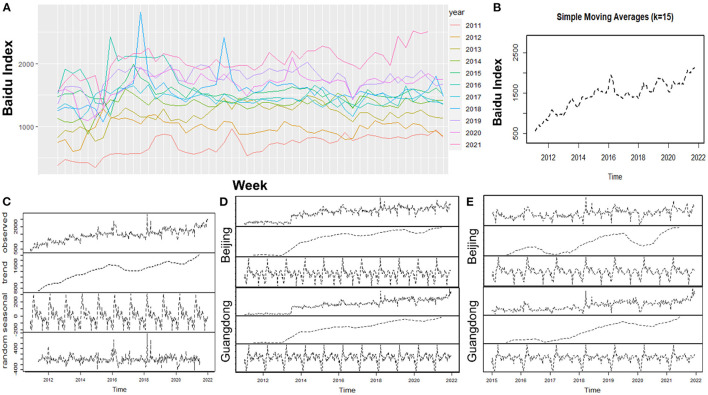
Trends and in seasonality of BI in Chinese mainland about DED: **(A)** Overall trends by weekly in different year, **(B)** Smoothing timeseries curves, **(C)** Decomposition timeseries curve, **(D)** Decomposition timeseries curves in Beijing and Guangdong and **(E)** Decomposition timeseries curves in Beijing and Guangdong from 2015 to 2021.

Overall, the BI of DED in Chinese mainland had gradually increased over time, and the smoothed timeseries curve indicated a rapid increase from 2012 to 2014, followed by a slow rise. In addition, a large random fluctuation can be observed during 2016 (Figures 1A,B). The trend, seasonality and random error were further decomposed from the timeseries curve, and it could also be seen the overall interest in DED was on the rise from the decomposition trend curve (Figure 1C). The regular seasonal curve indicated the existence of seasonality in searching DED, which could explain 42.66% of the variability in the timeseries curve. Furthermore, there also existed a significant random error in 2018 which was much higher than in 2016 from the decomposition random error curve.

We further selected two representative regions in the north (Beijing) and south (Guangdong) with the highest incidence of DED and the similar internet penetration rate to explore whether there is a difference in seasonality between different areas (Figures 1D,E). Both decomposition trend curves of them demonstrated s significant rise of BI from 2012 to 2014, as previously depicted in Figure 1C, and the two decomposition seasonal curves presented the BI index of DED gradually declined over the year and rose rapidly at the start of the following year, which implied the peak of BI about DED was in winter. However, their seasonality was only 0.29 and 0.17, respectively. In order to avoid the impact of the rapid rise in 2012 to 2014 on the decomposition of timeseries curve, we decomposed these timeseries curve of two cities for the period 2015 to present. The results identified the seasonality of Beijing was 0.50, and higher than that of Guangdong (0.23).

### Geographic Trends and Hotspots of DED

**Figure 2:**
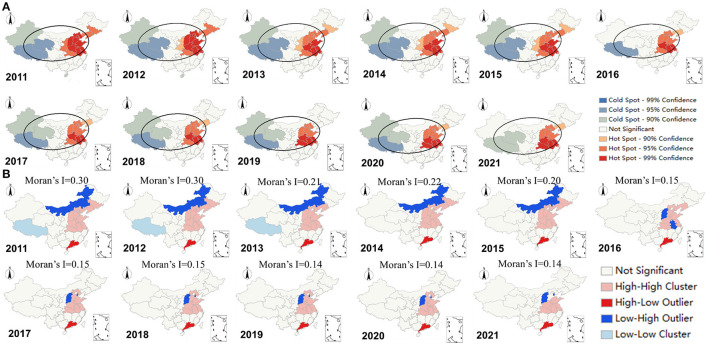
Spatial distribution of BI in Chinese mainland about DED: **(A)** Hotspot analysis and directional distribution, **(B)** Global (Moran's I index) and local spatial autocorrelation.

Figure 2A demonstrates the spatial hotspot and coldspot distribution of BI about DED. Most hotspots were concentrated in the east of Chinese mainland, while the coldspot mainly concentrated in west. In addition, the distribution of hotspots in the northeast and central regions gradually decreased, with the central region of Shaanxi province no longer being a hotspot for DED BI since 2016, so as the northeast region of Jilin province. The overall development trend of the DED BI was analyzed through standard deviation ellipse, which can be seen that the shape of the ellipse remained stable over the last decade, indicating that the DED BI always presented a distribution that gradually decreased from the east to the west.

The global Moran's I spatial autocorrelation analysis of the BI about DED in Chinese mainland from 2011 to 2021 was conducted at the provincial level (Figure 2B). The results showed that the Moran's I index was >0 for all years from 2012 to 2017 with statistical significance, however there also demonstrated an overall decreasing trend of Moran's I index, which indicated that the spatial aggregation of DED BI was gradually weakening. From 2018 onwards, the Moran's I index was no longer significant, indicating that DED BI began to take on the character of a random distribution instead of aggregation. We further analyzed the local spatial autocorrelation and its LISA aggregation map to explore aggregation areas of BI about DED in Chinese mainland. It could be seen that the High/High clustering was mainly concentrated on the eastern regions, while the High/Low clustering was concentrated on Guangdong province, which implied that the Guangdong was also a major agglomeration location of DED BI besides the eastern.

### Public Attention of DED

**Figure 3:**
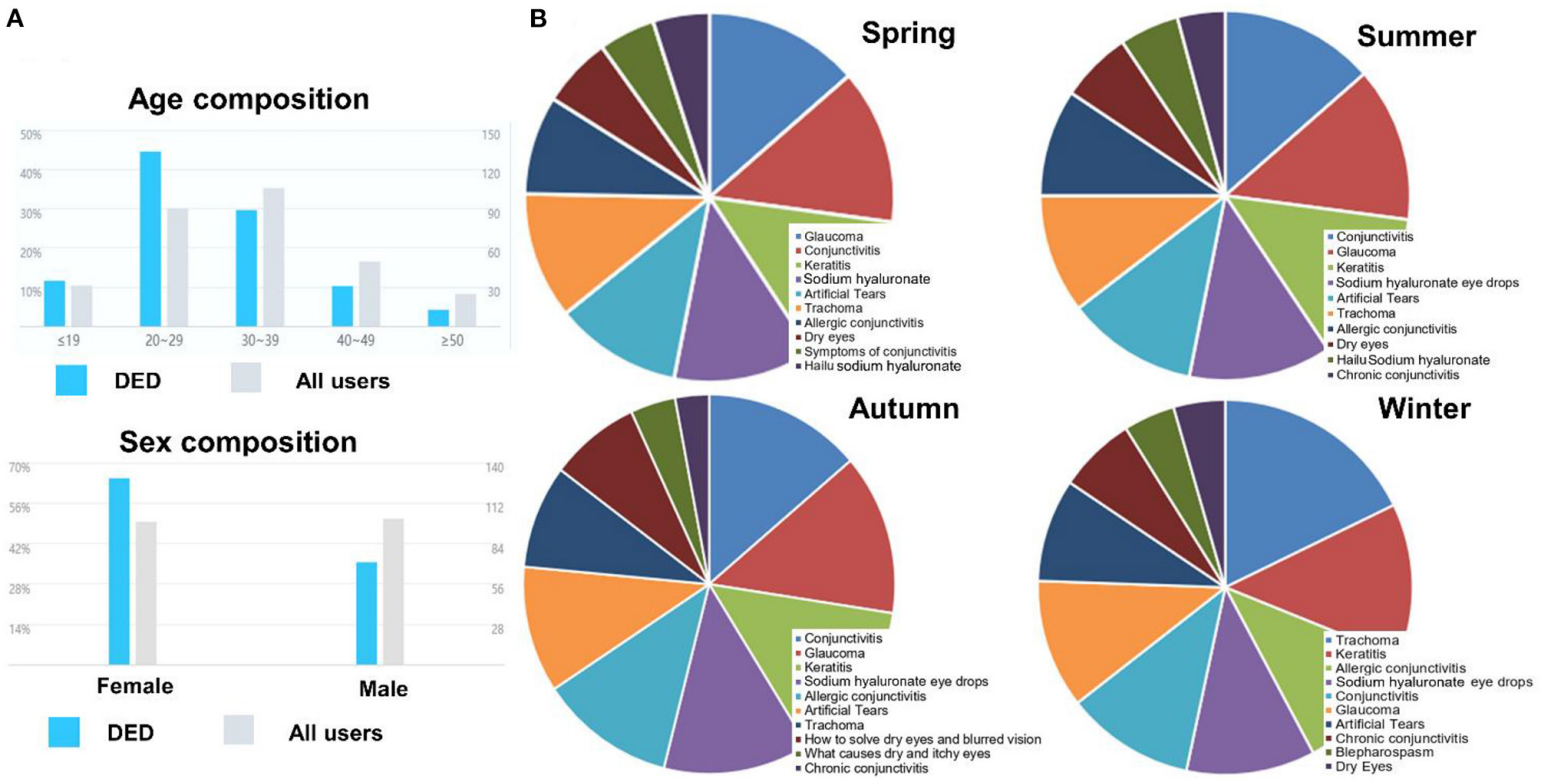
**(A)** Characteristics of users who searched DED in Baidu, **(B)** Distribution of DED-related terms in searching of different seasons during the last year (Top 10).

Figure 3A shows the characteristics of users who searched DED in Baidu, in which could observe that people aged between 20–29 years searched DED most, much more than the average of all Baidu users while people in 30 s were slightly less than the average levels. In addition, there was a higher proportion of women than men in searching for DED-related keywords. We further summarized the top 10 DED-related search keywords in the past year and divided them by season (Figure 3B). The ocular diseases were always at the top of the search list in different seasons, including glaucoma, conjunctivitis, keratitis and trachoma. This was followed by treatments for DED, such as eye drops, artificial tears and sodium hyaluronate (commercial name: Hailu). Furthermore, there were also many searches about symptoms including dry and itchy, especially in autumn.

## Discussion

The internet has become so widely involved in the daily lives of people, and the data from search engine can reflect public perceptions and interests of particular issues or diseases to some extent, especially when available data from large scale on-site epidemiological studies are lacking (18, 19). In this study, we identified search trends of DED in relation to seasonality, geographical hotspot distribution, and public concerns based on Baidu, the most widely used search engine in Chinese mainland. Such results could provide a meaningful reference for further understanding the epidemiology of DED in Chinese mainland and developing more appropriate interventions.

It is generally believed that the incidence and prevalence of DED is increasing, however, there is still lack of sufficient evidence to support this view (20). Our research provided circumstantial evidence through the trend of increasing BI in DED over the years in Chinese mainland. Among the timeseries curves, 2012–2014 and 2018 could be considered as the key burst time points of searching DED. The reason of a steeply rising BI about DED from 2012–2014 might be due to the widespread use of 4G phones, people started to use their phones more often for searching and the prolonged screen use could also cause more DED (21). During 2017–2018, DED had attracted a lot of attention from the researchers, including the top medical journal *The New England Journal of Medicine* published a thematic review, and the TFOS organized a series of workshop to revise the guidelines for diagnosis and treatment of DED (22–24). These efforts had contributed to the awareness and understanding of DED among clinicians and the general public.

Our research showed BI about DED varied seasonally, with an overall trend of high in winter and low in summer, which met the previous epidemiological studies (25–27). Such trend was more evident in the northern regions, while the southern was less affected by the seasons (4). Um et al. (28) and van Setten et al. (29) further proposed that this seasonality could be attributed to the triggering factors of DED symptoms in the environment like ambient temperature and humidity. Therefore, the low seasonality of Guangdong could be explained by it belongs to the subtropical monsoon climate, with the lower range of temperature variations and higher humidity compared with Beijing. However, Zhong et al. (30) reported the opposite conclusion, and they found the seasonality played an important role in prevalence of DED in Taiwan, which performed a similar geographical location to Guangdong. This might be due to regional differences in industrial structure, resulting in different lifestyles, which was a more important risk factor than the season under the same climatic conditions.

Previous epidemiological studies of DED in Chinese mainland showed that the overall prevalence was highest in eastern while lowest in northwest owing to the differences in climatic conditions and socioeconomic factors (4, 28). Our geographical hotspot analysis of the BI about DED led to the same conclusion, however this trend in geographical distribution had decreased in the last decade, as evidenced by the hotspot shift path and diminishing spatial autocorrelation. Such phenomenon also confirmed that the incidence of DED gradually increased, especially in the high-altitude western regions after 2014. In addition to the impact of high altitude that was identified as the risk factor of DED, the proliferation of mobile devices might also accelerate this process (31, 32). Moreover, we also identified the Guangdong province was a high-risk area of DED compared with surroundings by local autocorrelation, which was often overlooked in previous studies. This was probably due to the fact that Guangdong province was the place where the novel electronics and internet businesses developed most rapidly in Chinese Mainland (33, 34). At the same time, there was an influx of heavy screen users working in these areas each year, which could also explain the BI trend about DED gradually shift to the east and become concentrated (35). Furthermore, there are still lack of large epidemiological studies on screen use and DED prevalence due to the difficulties in data collection, while the population enrolled in this study usually had a habit of using electronic devices, which can provide a useful reference for the future studies.

User profile showed that women were the main searchers, which was consistent with previous research that women are at greater risk of DED (36). However, several studies reported the age was the important risk of DED, while we found the teenagers search more about DED than middle-aged and older in this study (37–39). This may be due to differences in the study population, with adolescents likely to be more adept at accessing information from the internet. In addition, adolescents might have subjective symptoms of DED but do not meet the actual diagnostic criteria for field epidemiological studies (40, 41). The frequency analysis of hot words related to DED mainly includes three aspects: other associated ocular diseases, treatment options and the subjective symptoms of patients. Keratitis, conjunctivitis, glaucoma, trachoma and refractive disorders are the hottest related searches about DED, and when patients feel uncomfortable with the eyes but do not know exactly about diagnosis, they tend to search such words owing to the semantic similarity in Chinese. This suggests that we should further raise awareness of these diseases for the public. The most frequent hot word of treatment is sodium hyaluronate and artificial tears, which is most common prescription for clinicians and also is over-the-counter medicines. Patients may be likely to search its side-effect and purchase it when receive the advice from doctors or netizen. In addition, patients also frequently search for their symptoms online, especially in the autumn, further suggesting that there is a seasonality to the prevalence of DED. These search behaviors indicate the propensity of patients to use the internet for medical consultations or to search for medical services, however the potential risks of suffering cyberchondria of excessive searching should be further concerned (42).

There are some limitations in this study. The result cannot directly reflect the prevalence of DED, because many people who simply feel dryness in eyes might also search “Dry eye diseases” in Baidu, while they do not actually meet the criteria for a diagnosis. In addition, the details in the process of generating and calculating BI is not disclosed by the Baidu Inc., and the BI only provided relatively little information and short time span compared to Google trends. Therefore, the demographic characteristics and potential covariates that impacted on the searching behavior of DED could not be further identified and analyzed in this research.

## Data Availability Statement

The original contributions presented in the study are included in the article/supplementary material, further inquiries can be directed to the corresponding author.

## Author Contributions

HY and YF designed the study. HY and WZ wrote the initial draft. MZ, GZ, WW, and YF revised the manuscript. All the authors made a substantial contribution to the article and approved the final submitted version.

## Funding

This study was supported by the National Natural Science Foundation of China grants (Nos. 81700799; 82070926).

## Conflict of Interest

The authors declare that the research was conducted in the absence of any commercial or financial relationships that could be construed as a potential conflict of interest.

## Publisher's Note

All claims expressed in this article are solely those of the authors and do not necessarily represent those of their affiliated organizations, or those of the publisher, the editors and the reviewers. Any product that may be evaluated in this article, or claim that may be made by its manufacturer, is not guaranteed or endorsed by the publisher.
